# Vacancy and Strain Effects on the Stability and Electronic Properties of 2D-Mg Intercalated GaN

**DOI:** 10.3390/ma18204755

**Published:** 2025-10-16

**Authors:** Qilin Wu, Shuqing Zhang, Xiaoyan Song, Xinping Zhang

**Affiliations:** 1 Institute of Information Photonics Technology, School of Physics and Optoelectronic Engineering, Beijing University of Technology, Beijing 100124, China; qlwu2001@emails.bjut.edu.cn (Q.W.); zhangxinping@bjut.edu.cn (X.Z.); 2 College of Materials Science and Engineering, Key Laboratory of Advanced Functional Materials, Ministry of Education of China, Beijing University of Technology, Beijing 100124, China; xysong@bjut.edu.cn

**Keywords:** gallium nitride, 2D-Mg intercalation, Ga vacancies, strain control

## Abstract

The recent discovery of two-dimensional Mg (2D-Mg) intercalation in GaN has attracted increasing attention, prompting fundamental questions regarding its structural stability and electronic properties. In this work, we employ first-principles calculations to investigate the structural and electronic effects of 2D-Mg intercalation in GaN. We identify the most energetically favorable intercalation ratio of Mg, reveal the critical role of Ga vacancies in restoring semiconducting behavior, and demonstrate that compressive strain further modulates the electronic structure. In particular, the configuration with 75% Mg intercalation and nearest-neighbor Ga vacancy under compressive strain exhibits significant band gap narrowing and enhanced Mg-related acceptor activity. These findings challenge long-standing assumptions about Mg clustering and establish a mechanistic framework based on intercalation, vacancy engineering, and strain control for the design of next-generation p-type GaN devices.

## 1. Introduction

Gallium nitride (GaN) has emerged as a critical wide-bandgap semiconductor for advanced power electronics and optoelectronic devices, owing to its excellent intrinsic properties [1,2,3,4]. However, achieving effective p-type doping remains a major challenge that limits further performance improvements [5,6]. Mg is currently the only practical p-type dopant for GaN and has been applied in commercial optoelectronic devices, such as blue LEDs [7,8]. Nevertheless, the realization of high-performance p-type GaN remains far from ideal [9]. The deep acceptor level of Mg leads to low ionization efficiency at room temperature [10], while compensation from native defects significantly limits hole concentration and mobility [11]. These intrinsic limitations pose a critical bottleneck to the development of next-generation high-efficiency p-type GaN devices.

To address these issues, various alternative strategies have been proposed, including co-doping with additional impurities to enhance acceptor ionization [12,13], polarization-induced doping to exploit built-in electric fields in heterostructures [14], and strain engineering to modify the band structure and enhance hole transport [15,16,17]. While these approaches have yielded incremental improvements, they often involve complex fabrication processes or provide limited control over hole mobility and transport. Moreover, when Mg doping concentrations exceed ~10^19^ cm^−3^, closed pyramidal inversion domains tend to form, further degrading hole conductivity due to disrupted crystal polarity and defect clustering [17,18,19].

Intercalation has long been recognized as an effective route to tune structural stability and electronic properties of materials [20]. Recently, a fundamentally different strategy has emerged based on the intercalation of two-dimensional Mg (2D-Mg) layer into GaN, forming so-called Mg-intercalated GaN superlattices (MiGs). The feasibility of such superlattice formation is further supported by the close lattice match between GaN and Mg, which minimizes interfacial strain and stabilizes the intercalated configuration. Experimental studies have shown that samples containing MiGs exhibit up to a six-fold enhancement in hole transport compared to non-intercalated samples, primarily attributed to strong out-of-plane compressive strain and periodic polarity inversion. However, despite these experimental advances [21], the fundamental mechanisms underlying the structural stability and electronic modulation induced by 2D-Mg intercalation remain poorly understood. Our study addresses this gap by providing a systematic theoretical investigation. 

In this work, we systematically investigate the influence of 2D-Mg intercalation on the structural and electronic properties of GaN using first-principles calculations. We construct a series of intercalated models with varying Mg coverage, compare their formation energies, and identify the most stable configuration. Introducing Ga vacancies near the Mg layer to maintain charge neutrality not only restores semiconducting behavior but also generates shallow Mg acceptor states. Additionally, compressive strain along the *c*-axis further narrows the band gap and facilitates hole excitation. These results provide mechanistic insights into the role of intercalation, vacancies, and strain in modulating the electronic properties of GaN.

## 2. Materials and Methods

**Figure 1:**
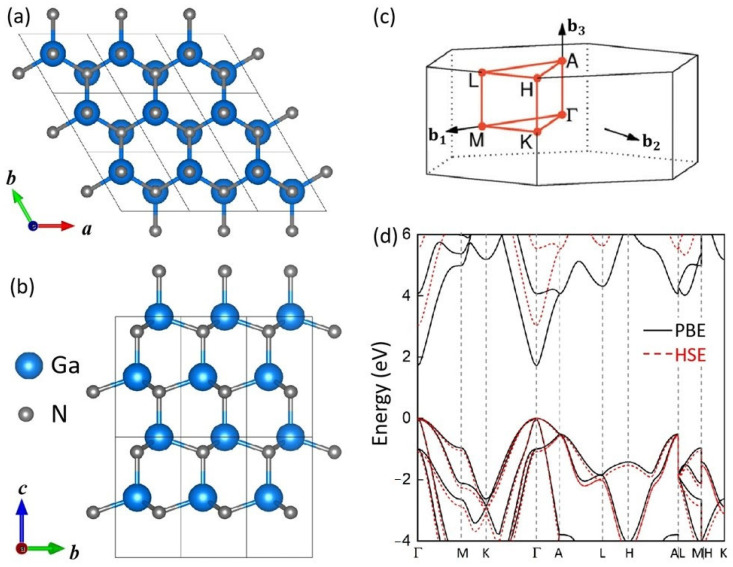
(**a**) Top and (**b**) side views of the atomic structure of GaN. (**c**) The first Brillouin zone for bulk GaN. (**d**) Band structure of GaN calculated by PBE and HSE functionals.

First-principles calculations are performed using projector augmented wave (PAW) method [22] as implemented in the Vienna ab initio Simulation Package (VASP) [23,24]. Structural optimization was completed until the total energy and atomic forces converged to 10^−5^ eV and 0.01 eV/Å, respectively. A plane-wave energy cutoff of 500 eV was used, and the Brillouin zone of unit cell was sampled with a Monkhorst–Pack k-point mesh [25] of 15 × 15 × 9, corresponding to a maximum spacing of 0.02 Å^−1^. The relaxed atomic structure of hexagonal GaN is shown in Figure 1a,b. In all intercalated models, a vacuum layer larger than 15 Å was included along the *c*-axis to avoid spurious interactions. Uniaxial compressive strain was applied along the [0001] direction, ranging from 0% to −10% in steps of −2%. The expressions used to calculate the formation energies are provided in the Supporting Information.

To assess the reliability of the exchange–correlation functional, we compared the Perdew–Burke–Ernzerhof (PBE) functional [26] with the Heyd–Scuseria–Ernzerhof (HSE06) hybrid functional [27] by calculating the band structure of GaN unit cell. As shown in Figure 1c,d, PBE significantly underestimates the band gap, as expected. However, the band-edge exhibit strong agreement with HSE06, and the calculated electron and hole effective masses according to the band edges are in close correspondence for both functionals; the detailed comparation is shown in Table S1 in the Supplementary Materials. Notably, the valence band dispersion obtained from PBE nearly overlaps with that of HSE06. Since this work primarily focuses on hole-related properties, and in view of the substantial computational cost of hybrid functionals, we adopted the PBE functional for all subsequent calculations.

## 3. Results

**Figure 2:**
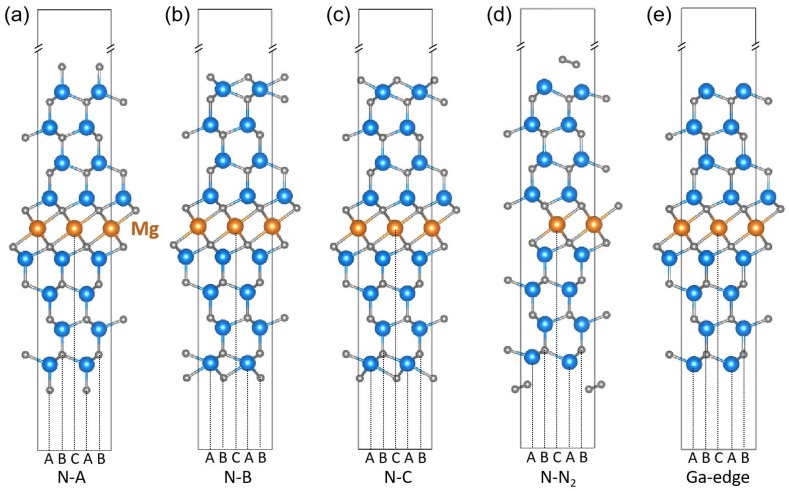
(**a**–**e**) Schematic illustration of five possible edge configurations for 2D-Mg intercalated GaN slab models.

To investigate the structural and electronic properties of 2D-Mg intercalated GaN, we first constructed models consisting of GaN slabs separated by a monolayer of Mg, as illustrated in Figure 2. Bulk GaN crystallizes in a hexagonal structure with an ABBA stacking sequence and exhibits strong spontaneous polarization along the [0001] (*c*-axis) direction. In our model, a single Mg atomic layer is inserted at the central C-site position, with GaN slabs placed on both sides in a centrosymmetric configuration. This arrangement leads to opposing polarization directions in the two GaN regions with respect to the Mg layer, resulting in an inversion-symmetric structure about the Mg plane that cannot be periodically extended along the *c*-axis. To resolve this, we constructed finite slab models with symmetric truncation at both ends. This modeling strategy is consistent with earlier theoretical work. For example, Northrup et al. [17] analyzed possible Mg incorporation configurations at inversion domain boundaries in GaN using boundary-based slab models, underscoring the necessity of non-periodic approaches to capture polarity inversion effects.

We then investigated various edge terminations by evaluating their relative stabilities. Specifically, we considered nitrogen-terminated edges with the outermost N atoms occupying the A-, B-, or C-sites, as well as a reconstructed nitrogen edge where surface N atoms form molecular N_2_ dimers. These models are referred to as the N-A, N-B, N-C, and N-N_2_ configurations, respectively, as shown in Figure 2. In addition, a Ga-terminated edge (Ga-edge) was also modeled for comparison.

The formation energies of the edge configurations in Figure 2 are summarized in Table 1. Among the nitrogen-terminated models, only the N_2_-terminated edge exhibits a negative formation energy, indicating its thermodynamic favorability. The desorption energy of the N_2_ dimer was calculated to be 0.11 eV using the climbing image nudged elastic band method [28] (see Figure S1 in the Supplementary Materials). This low desorption barrier falls within the typical range of physisorption energies and suggests that N_2_ can readily desorb under mild thermal conditions [29]. Following N_2_ desorption, the resulting structure corresponds directly to the Ga-terminated configuration. Notably, the Ga-edge structure also shows a negative formation energy, confirming its stability. Therefore, owing to the ease of N_2_ desorption and the favorable energetics of the Ga edge, we adopted the Ga-terminated configuration in all subsequent calculations to ensure both thermodynamic plausibility and structural consistency.

To further understand the electronic characteristics of various edge configurations, we calculated the projected density of states (PDOS) for the five edge structures discussed above, as presented in Figure S2 of the Supplementary Materials. In all cases, the occupied Mg states are located below –3 eV, indicating that Mg in the fully 2D intercalated configuration is unlikely to serve as an effective acceptor for hole conduction. Furthermore, due to the metallic nature of the 2D-Mg layer itself, the density of states exhibits metallic characteristics across all five configurations. Experimental studies also suggest that Mg atoms are not completely intercalated at the C-sites in a continuous fashion. Instead, localized 2D-Mg sheets are typically embedded between GaN layers. 

**Figure 3:**
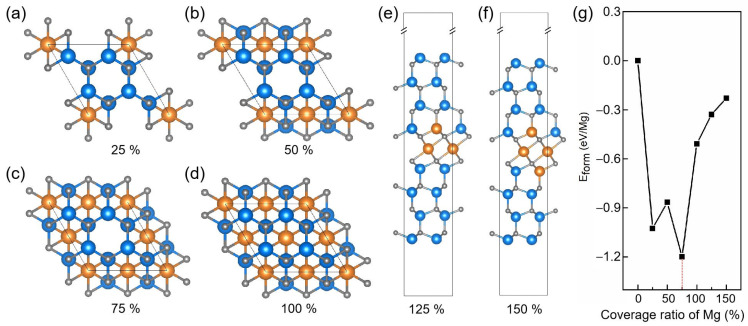
(**a**–**d**) Top views of 2 × 2 GaN supercells with Mg intercalation ratios of 25%, 50%, 75%, and 100%, respectively. (**e**,**f**) Side views of extended configurations with Mg coverage of 125% and 150%, where excess Mg atoms substitute neighboring Ga sites. (**g**) Formation energies of all models as a function of Mg coverage ratio.

To evaluate the impact of Mg intercalation ratio on the structural stability, we constructed a series of models with nominal Mg coverage of 25%, 50%, 75%, 100%, 125%, and 150% within a 2 × 2 GaN supercell, as illustrated in Figure 3a–f. The 125% and 150% models correspond to extended configurations in which Mg atoms not only form a complete monolayer but also substitute adjacent Ga atoms, in line with experimental observations of partial Mg diffusion into Ga sites [21]. The calculated formation energies reveal that the 75% intercalation model is the most energetically favorable among all models as shown in Figure 3g. This configuration was therefore selected as the basis for subsequent analysis.

The PDOS for these models are presented in Figure S3. The 25% and 50% intercalation structures retain semiconducting characteristics, whereas configurations with 75% Mg coverage or higher display metallic states near the Fermi level, indicating a transition from semiconducting to metallic behavior with increasing Mg concentration. In practical devices, such metallicity may provide leakage pathways and reduce efficiency.

To explore possible mechanisms for mitigating this metallicity, we next considered the role of Ga vacancies near the Mg layer. This choice is motivated both by the need to compensate for the excess electrons introduced by intercalated Mg atoms and by experimental observations showing that the Ga signal intensity adjacent to the Mg layer decreases to approximately 75% of its original value [17,21], consistent with partial Ga depletion. Accordingly, Ga vacancies were introduced into our models to examine their compensating effect and impact on electronic properties. 

**Figure 4:**
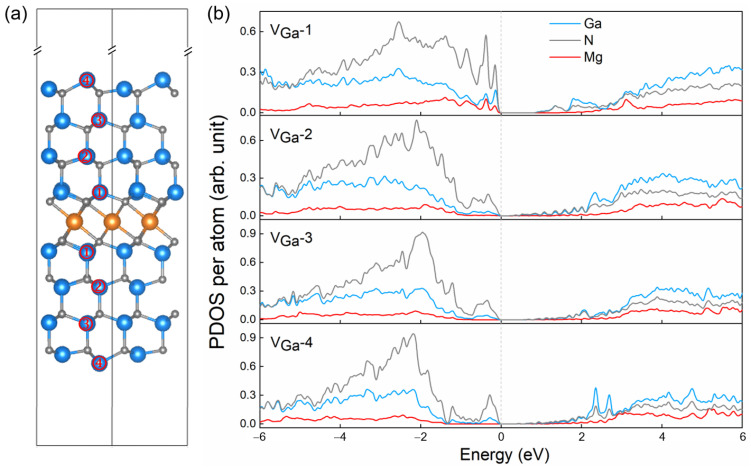
(**a**) Side view of the 75% Mg intercalation model, showing the positions of Ga vacancies introduced at four different sites: first-nearest (V_Ga_-1), second-nearest (V_Ga_-2), third-nearest (V_Ga_-3), and outermost-layer Ga (V_Ga_-4) relative to the Mg layer. (**b**) PDOS per atom for the four Ga-vacancy configurations.

For the thermodynamically most stable 75% Mg coverage model (i.e., three Mg atoms contributing six excess electrons in a 2 × 2 supercell), we removed two Ga atoms to achieve charge compensation. These vacancies were introduced symmetrically, with one located above and the other below the Mg layer. Four distinct vacancy configurations were considered, denoted as V_Ga_-1 through V_Ga_-4, corresponding to the removal of Ga atoms at first-nearest, second-nearest, third-nearest, and outermost-layer positions relative to the Mg layer, respectively, as illustrated in Figure 4a.

The electronic structures of the four Ga-vacancy configurations (V_Ga_-1 to V_Ga_-4) under 75% Mg coverage were further analyzed by calculating their PDOS, as shown in Figure 4b. All four configurations exhibit semiconducting behavior, in sharp contrast to the metallic character of the defect-free intercalated structure (see Figure S3 for details). The calculated PBE bandgaps are 0.98, 0.32, 0.65, and 0.39 eV for V_Ga_-1 through V_Ga_-4, respectively. The introduction of Ga vacancies not only restores the semiconducting nature of the system but also reduces the bandgap relative to pristine GaN, thereby lowering the thermal activation energy required for hole conduction. In particular, the V_Ga_-1 configuration, where Ga vacancies are located nearest to the Mg layer, exhibits pronounced Mg-related states near the valence band maximum, suggesting an increased likelihood of Mg atoms acting as effective acceptors. This configuration also aligns with experimental observations reporting an approximately 25% reduction in Ga signal intensity near the Mg layer [21]. According to the Bader analysis, each Mg atom loses approximately 1.6 e^−^ for the VGa-1 configuration, with the excess charge mainly transferred to the neighboring N atoms. This indicates that Mg sites are electronically depleted, which is consistent with the emergence of Mg-related unoccupied states near the valence band maximum observed in the PDOS.

**Figure 5:**
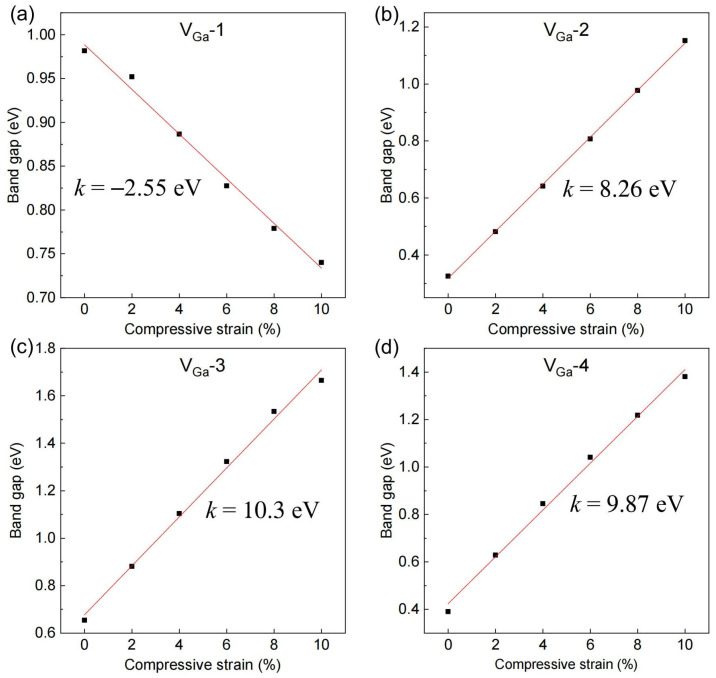
(**a**–**d**) Bandgap evolution under uniaxial compressive strain along the GaN [0001] direction for the four Ga-vacancy configurations (V_Ga_-1 to V_Ga_-4).

Motivated by experimental observations indicating that 2D-Mg intercalation can induce compressive lattice strain as high as 10% along the [0001] direction of GaN [21], we systematically investigated the impact of uniaxial compression strain applied along the *c*-axis on the bandgap evolution of the four Ga-vacancy configurations, as shown in Figure 5. For V_Ga_-2 through V_Ga_-4, the bandgap increases with strain, consistent with the typical behavior of conventional semiconductors such as Si, Ge, and GaN [30,31]. In contrast, the V_Ga_-1 configuration exhibits a distinct trend: its bandgap decreases monotonically under compressive strain. This unusual response suggests enhanced transport, as the reduced bandgap lowers the thermal activation barrier for carrier excitation. Notably, at 10% compressive strain, the bandgap of V_Ga_-1 is reduced to 0.74 eV, which is less than half the intrinsic PBE bandgap of unstrained GaN. Experimental studies on Mg-doped GaN thin films also emphasize the interplay between defect complexes and mechanical characteristics [32], supporting the relevance of strain- and defect-assisted mechanisms.

## 4. Conclusions

In summary, we have systematically investigated the structural and electronic properties of GaN systems with 2D-Mg intercalation using first-principles calculations. To model experimentally relevant configurations, truncated slab models with various edge terminations were constructed, and Ga-terminated edges were found to be thermodynamically favorable following N_2_ desorption from nitrogen-terminated surfaces. We further explored a series of partially intercalated models with varying Mg coverage ratios, and identified that the 75% intercalation model exhibited the lowest formation energy, identifying it as the most energetically favorable structure. To maintain charge neutrality and recover semiconducting behavior, symmetric Ga vacancies were introduced near the Mg layer. Among the four vacancy configurations studied, the nearest-neighbor Ga-vacancy structure (V_Ga_-1) not only opened a finite bandgap but also generated Mg-related acceptor states near the valence band maximum, which may enhance hole conductivity. Motivated by experimental reports of high elastic compressibility in such layered systems, we further examined the effect of uniaxial compressive strain along the *c*-axis. The VGa-1 configuration displayed an anomalous bandgap under increasing compression, potentially facilitating hole excitation. 

Overall, this study provides mechanistic insight into the doping behavior of 2D-Mg intercalated GaN and its potential for improved p-type functionality, offering theoretical guidance for future experimental design of high-performance p-type GaN heterostructures.

## Figures and Tables

**Table 1:** Formation energies of five edge configurations in Figure 2.

Edge Configuration	N-A	N-B	N-C	N-N_2_	Ga-Edge
*E*_form_/eV	6.48	5.02	5.62	−0.63	−0.51

## Data Availability

The original contributions presented in this study are included in the article. Further inquiries can be directed to the corresponding author.

## Supplementary Materials

The following supporting information can be downloaded at: https://www.mdpi.com/article/10.3390/ma18204755/s1, Table S1: Calculated electron and hole effective masses of GaN using PBE and HSE functionals.; Figure S1: Energy profile as a function of the distance between the N_2_ dimer and the GaN surface in the N–N_2_; edge configuration, calculated using the climbing image nudged elastic band (CI-NEB) method; Figure S2: The projected density of states (PDOS) per atom for the five edge structures discussed in Figure 2 in the text. Figure S3: Projected density of states (PDOS) per atom for models with different Mg intercalation ratios corresponding to those in Figure 3. Figure S4. Bandgap variation of the four Ga-vacancy configurations (VGa-1 to VGa-4) under uniaxial c-axis compression and coupled biaxial strain, where perpendicular *ab*-plane relaxation is considered via the Poisson ratio (ν = 0.18) of GaN.
