# Supplementary material for: Vacancy and Strain Effects on the Stability and Electronic Properties of 2D-Mg Intercalated GaN
Source: Materials (Basel). 2025 Oct 16;18(20):4755. doi: 10.3390/ma18204755 (PMC12565984; doi:10.3390/ma18204755)
Supplement: Supplementary file 1 [file materials-18-04755-s001.zip › materials-3910152-supplementary.pdf]

Supplementary Material

Enhanced hole conductivity in gallium nitride with 2D-magnesium-intercalation and gallium vacancy

Qilin Wu [1], Shuqing Zhang [1,*], Xiaoyan Song [2] and Xinping Zhang [1]

[1] Institute of Information Photonics Technology, School of Physics and Optoelectronic Engineering, Beijing University of Technology, Beijing 100124, People's Republic of China; qlWu2001@emails.bjut.edu.cn (Q. W.); zhangxinping@bjut.edu.cn (X. Z.)

[2] College of Materials Science and Engineering, Key Laboratory of Advanced Functional Materials, Ministry of Education of China, Beijing University of Technology, 100124, Beijing, China; xysong@bjut.edu.cn(X. S.)

* Correspondence: zhangsq@bjut.edu.cn (S. Z.)

Table S1. Calculated electron and hole effective masses of GaN using PBE and HSE functionals.

$m^*(m_0)$	PBE	HSE
$m_e^{\parallel}$	0.18	0.20
$m_{e1}^{\perp}$	0.28	0.28
$m_{e2}^{\perp}$	0.32	0.31
$m_h^{\parallel}$	2.11	2.42
$m_{h1}^{\perp}$	2.2	2.38
$m_{h2}^{\perp}$	1.69	1.47

Note: superscript "⊥" and "||" denote directions perpendicular and parallel to the [0001] (z-axis), respectively. Subscript 1 indicates values obtained along the Γ − M direction, and superscript 2 corresponds to those calculated along the K − Γ direction, both evaluated near the Γ point.

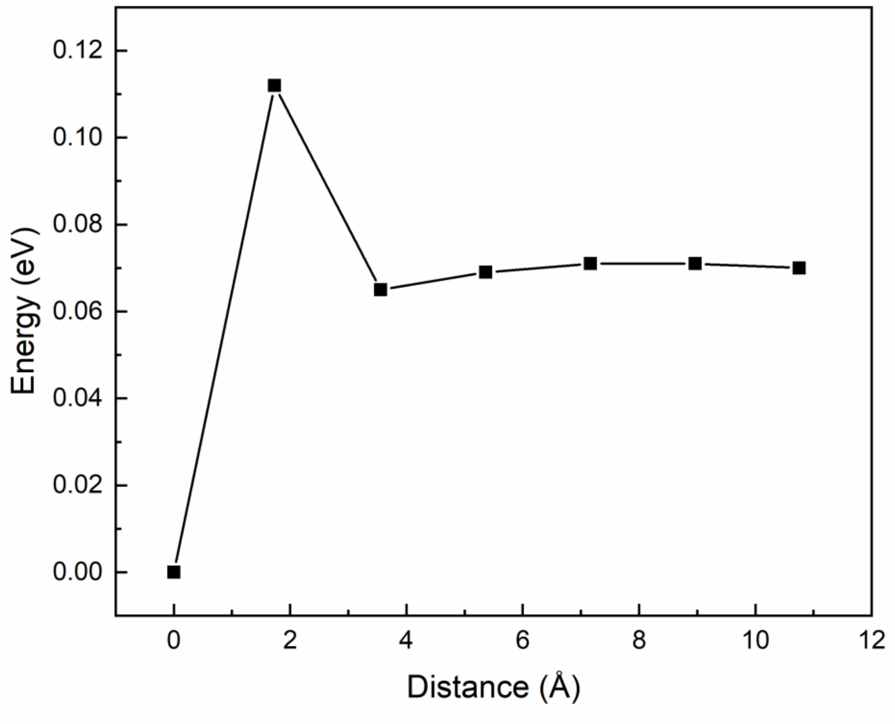

Figure S1. Energy profile as a function of the distance between the N_2 dimer and the GaN surface in the N–N_2 edge configuration, calculated using the climbing image nudged elastic band (CI-NEB) method.

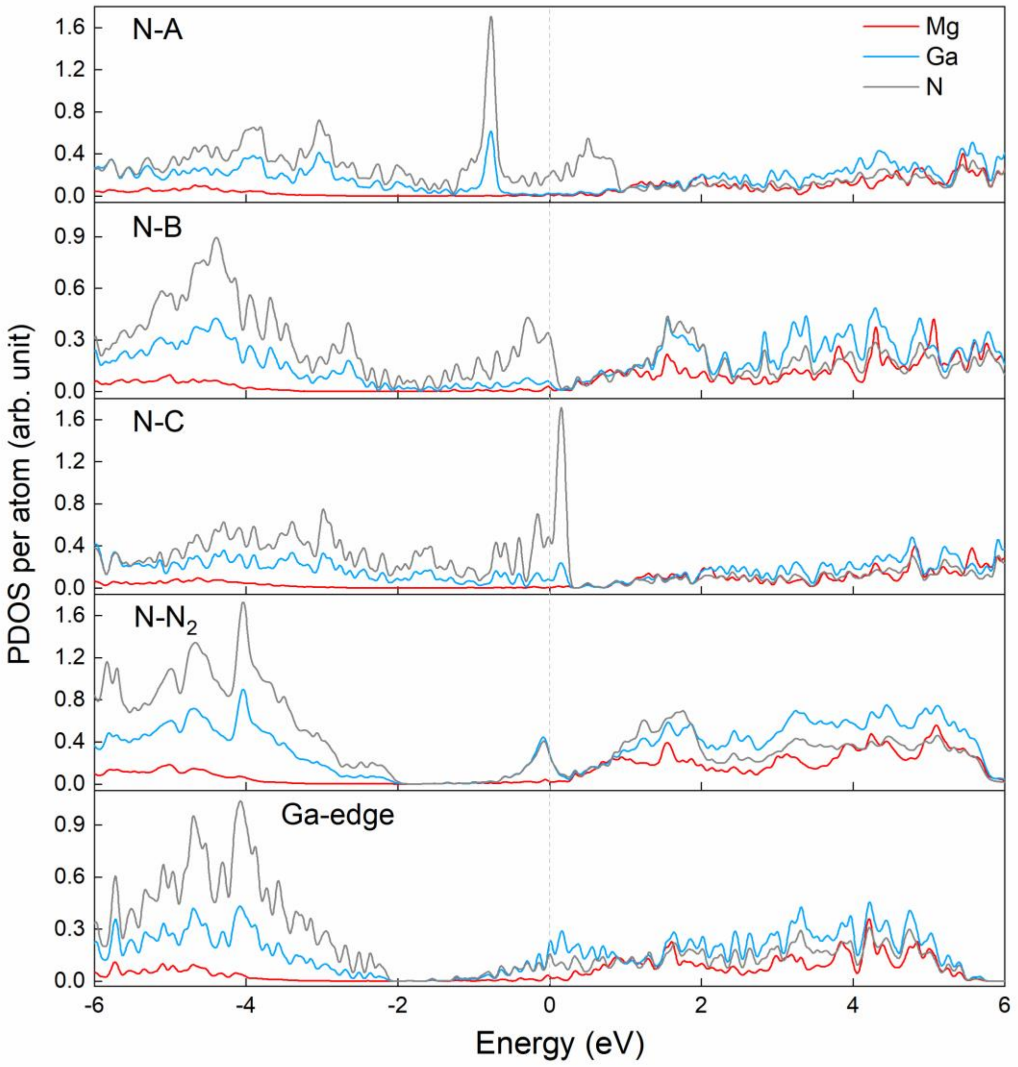

Figure S2. The projected density of states (PDOS) per atom for the five edge structures discussed in Fig. 2 in the text.

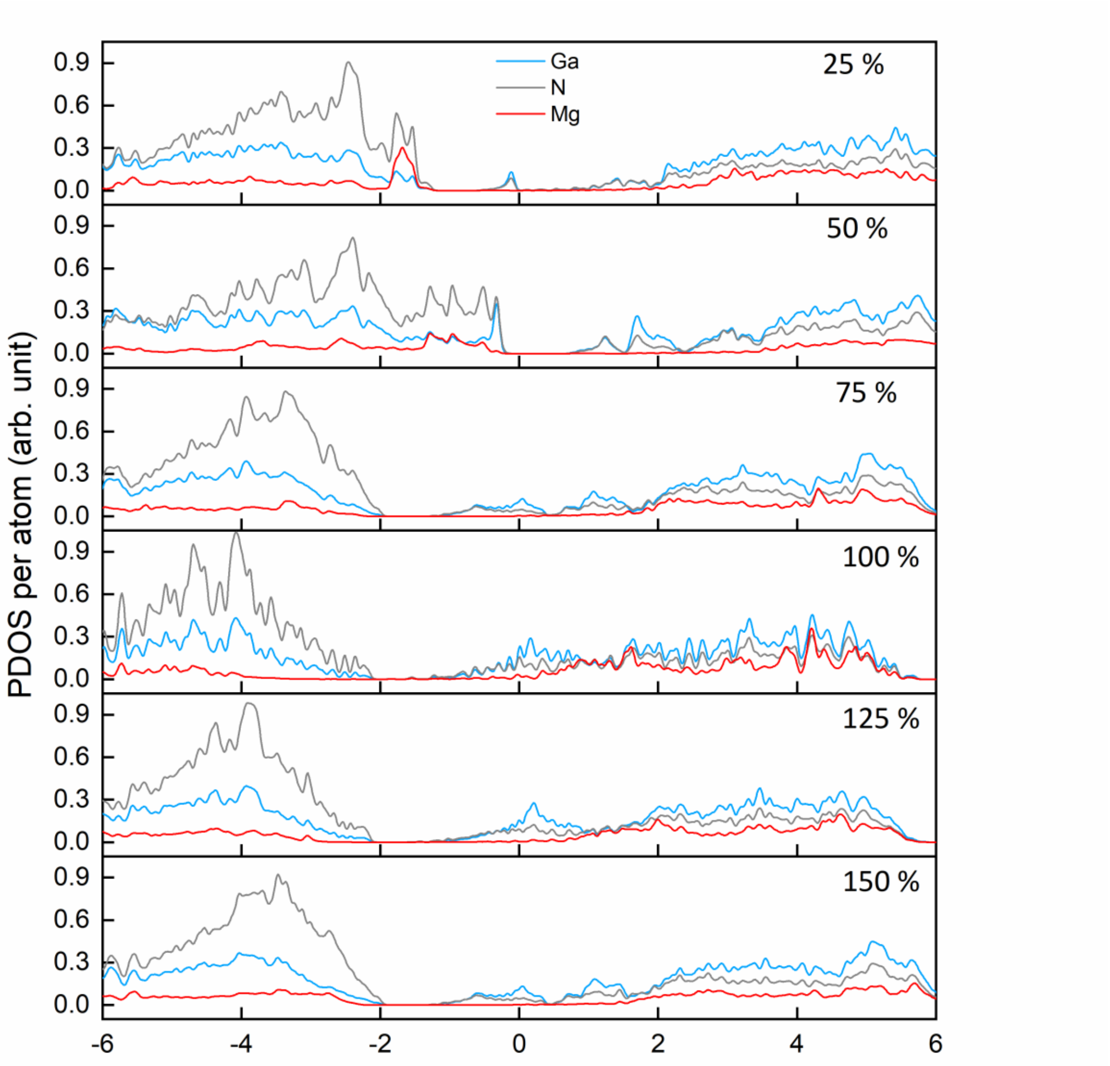

Figure S3. Projected density of states (PDOS) per atom for models with different Mg intercalation ratios corresponding to those in Fig. 3. While the 25% and 50% intercalation models retain semiconducting behavior, all structures with 75% Mg coverage or higher exhibit metallic characteristics, indicating a transition from insulating to metallic states with increasing Mg concentration.

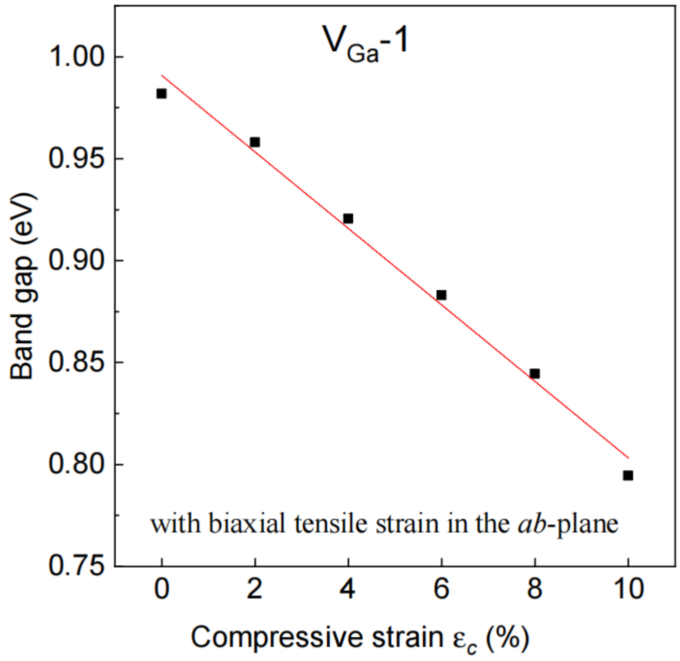

Figure S4. Bandgap variation of the V_{Ga}-1 configurations under uniaxial *c*-axis compression and coupled biaxial strain, where perpendicular *ab*-plane relaxation is considered via the Poisson ratio (ν = 0.18) of GaN. The biaxial strain was applied as simultaneous tensile deformation along both the x and y directions. The qualitative trend of bandgap narrowing remains unchanged, confirming that the main conclusions are robust with respect to the strain model.

S4: Computational equation of formation energy of Table 1 and Fig. 3g

The formation energies of the five edge configurations listed in Table 1 were calculated using the following expression:

$$E_1 = E_{edge} - n_1 E_{GaN} - \frac{1}{2} n_2 E_{N_2} - n_3 E_{Mg} \qquad (1)$$

where E_{edge} is the total energy of the edge model, E_{GaN} is the energy per GaN unit cell obtained from slab models used as the reference structure, E_{N_2} is the energy of an isolated N_2 molecule, and E_{Mg} is the energy per atom of bulk magnesium. n_1, n_2 and n_3 represent the number of GaN unit cells, surface nitrogen atoms, and intercalated Mg atoms, respectively.

The formation energies of the intercalated models in Fig. 3(a–f), as a function of Mg coverage ratio (Fig. 3g), were calculated using the following equation:

$$E_2 = E_{intercalated} - n_1 E_{GaN} - n_3 E_{Mg} \qquad (2)$$

where $E_{intercalated}$ is the total energy of the Mg-intercalated model.
